# An experience with mesh versus darn repair in inguinal hernias

**DOI:** 10.12669/pjms.333.13257

**Published:** 2017

**Authors:** Gulshan Ali Memon, Syed Kashif Ali Shah, Habib-ur-Rehman

**Affiliations:** 1 Gulshan Ali Memon, FRCS, MS. Professor and Dean Surgery and Allied, Department of General Surgery, Peoples University of Medical and Health Sciences for Women (PUMHS), Nawabshah, Sindh, Pakistan; 2 Syed Kashif Ali Shah, MS. Senior Registrar, Department of General Surgery, Peoples University of Medical and Health Sciences for Women (PUMHS), Nawabshah, Sindh, Pakistan; 3 Habib-ur-Rehman, FCPS. Assistant Professor, Department of General Surgery, Peoples University of Medical and Health Sciences for Women (PUMHS), Nawabshah, Sindh, Pakistan

**Keywords:** Inguinal / groin hernia, Lichtenstein procedure, suture repair, Bassini repair

## Abstract

**Background & Objective:**

The inguinal hernia accounts for 50 percent in old age males. A Lichtenstein type of operation has now become the method of choice in most developed countries but in the developing world traditional simple suture repair is still in common practice in resource limited hospitals due to the scarcity and expensive nature of the commercial prosthetic mesh. Our objective was to compare the rates of complications in Lichtenstein repair to tension free Darn repair.

**Methods:**

Ninety two male patients from 20-60 years of age reported for direct or indirect inguinal hernia with open Mesh/Lichtenstein or darn repair in emergency or electively from January 2014 to December 2015 were enrolled in this prospective randomized control trial (RCT). The primary end point was to compare the surgical site infection, length of hospital stay and hernia recurrence with different techniques.

**Results:**

The hospital stay was higher in patients who had Lichtenstein repair, Superficial surgical site infections in cohort A (6.5%) and cohort B (4.36%)were noted. Complications of recurrence in Group-A were (1.5%) as compared to Group-B which had a recurrence of 6.52%.

**Conclusion:**

Lichtenstein is more promising in comparison to Darn repair in terms of recurrence in inguinal hernia.

## INTRODUCTION

Among acquired or congenital hernias, inguinal remains to be most common, with prevalence of 25% and 2% in males and females respectively.^1^ The literature reports increased prevalence of inguinal hernia in old age and it accounts for 50 percent in elderly males annually.^2^ Inguinal hernia repair is the most common surgical procedure performed in the United States, with >600,000 performed on an annual basis.^3^ This intervention puts the highest burden on health care system.^4^ Despite technical advancements during last decades, the recurrence rates still remain as high as 15%.^5^ Lichtenstein, in 1987, introduced mesh prosthesis to bridge the hernia but not close it with sutures like the Bassini and other procedures. This ostensibly results in a less painful operation and a reduced incidence of suture pulling out, which results in lower recurrence rate.^6^,^7^

Gold standard for hernia repair all over the world now remains the use of polypropylene meshes as prosthesis.^8^ A Lichtenstein type of operation has now become the method of choice in most developed countries of the world. In developing countries traditional Bassini operation is still performed in resource limited hospitals due to the scarcity and expensive nature of the commercial prosthetic mesh.^9^ Light-weight PP meshes reduces the incidence of chronic groin pain as well as the risk of developing other groin symptoms.^10^ To avoid complications, the use of absorbable meshes – such as those made of lactic acid polymer or lactic and glycolic acid copolymers has been proposed. This exposes the patient to inevitable hernia recurrence because the inflammatory response, through a hydrolytic reaction, completely digests the implanted prosthetic material.^11^,^12^ After the introduction of tension-free surgical repair with the use of prosthetic mesh, recurrence rates are reported to be <5%, and patient comfort has also substantially improved compared with the traditional, tension-producing techniques.^13^,^14^ For synthetic mesh repairs many studies have noted their association with numerous complications, including persistent pain, infection, adhesions, bowel erosion, shrinkage, and inflammation.^15^,^16^

Our objective was to compare the complications in Lichtenstein repair with tension free Darn repair. We also looked at the surgical site infections, length of hospital stay, time taken to return to normal routine and recurrence of hernia.

## METHODS

Ninety two male patients between the age of 20-60 years who reported for direct or indirect inguinal hernia repair with open herniotomy and hernioplasty or herninorrhaphy (Mesh repair (Lichtenstein) or darn repair) in emergency or electively from January 2014 to December 2015 were enrolled in this prospective randomized control trial (RCT). Every patient gave informed consent when information about this study was provided. Patients having ASA class IV or above, malignancy or gangrenous bowls as content of sac, recurrent inguinal hernia or ascites were excluded. The patients were divided into two groups (Group A and group B) using lottery method before surgical incision. In group A patients were treated with mesh repair and in group B they were treated with darn repair.

In both groups Prophylactic antibiotic (cefotaxime) 1gm was administered half hour before surgery followed by two further doses 12 hourly after surgery in all the study patients. All patients had skin scrubbing with alcoholic chlorohexidine on operative table.

### Group A

Hernioplasty / Lichtestein tension free mesh repair. The subcutaneous fat and Scarpa Fascia was incised in line of incision and external oblique aponeurosis was separated in lines of it’s fibers to expose and deliver spermatic card. Sac was identified, negotiated, inverted, resected and ligated. Synthetic mesh was placed in posterior wall of the inguinal canal and fixed to the edges of defect with prolene sutures to have some tension free laxity and was irrigated with normal saline before closure of the inguinal canal in layers.

### Group B

These patients had Darn Repair in tension free continuous prolence (0-1) suture between the conjoined tendon and inguinal ligament with apposition between these structures. The same surgical team operated upon all these patients. The patients were discharged once their condition became satisfactory.

### Follow up

First visit was on 10^th^ day after discharge, then they were followed up every month for one year. The patients were only examined clinically by study team. Immediate post-operative (within 14 days), early post-operative (within 6 weeks) and late post-operative (within one year) complications of both procedures were recorded. Statistical analysis was performed using SPSS software version 18.01 (SPSS Inc. Chicago Illinois) for windows, ordinal variables were analyzed using x^2^ test, nominal variables were analyzed with fisher exact test.

## RESULTS

Ninety two male patients were enrolled in this study. Demographics characteristics i.e. age, BMI, ASA score, comorbidities, types of inguinal hernia, in A & B cohorts are shown in Table-I.

**Table-I:** Demographic Characteristics of patients in both the groups.

*Characteristics*	*Group A*	*Group B*
Patients enrolled	46	46
Age (Mean, years)	52.3	54.3
Range	(22-58)	(20-60)
ASA (n%) I	30	32
ASA (n%) II	16	14
Controlled Diabetes Mellitus	07	12
COPD	07	06
History of smoking	18	20
R.I.H (Indirect)	21	20
L.I.H (Indirect)	10	11
Direct	07	08
Bilateral	08	07
Elective surgery	34	32

**Table-II:** Outcome in both the groups.

*Characteristics*	*Group A*	*Group B*
SSI / Superficial	03	02
SSI / Deep	00	01
Length of hospital stay	(2-14)	(2-4)
Time taken to return to daily activities	(14-28)	(14-21)
Hernia recurrence	01	03

There were no significant differences in both the groups as regards age, BMI, ASA score in both groups. Almost equal number of patients from both cohorts were operated in emergency or electively. However, numbers of Diabetic patients were little more in cohort A. there was no difference in intensity and duration of pain in both groups postoperatively up to two weeks. However, the hospital stay was higher in patients who had Lichtenstein repair. Superficial surgical infections in cohort A was 6.5% as compared to 4.36% in cohort B. Complication of recurrence in group A was 1.5% as compared to 6.52% in Group-B.

## DISCUSSION

After appendectomy, repair of inguinal hernia is the most common (10-15%) surgical procedure performed all over the world.^17^ Since the Bassini’s repair in 1887, numerous operative techniques have been reported,^18^ but yet no definitive operative technique is considered the best. The material used remains controversial. Some of these techniques are very much practiced today while some have become obsolete. In spite of these ever changing trend of techniques, surgical acumen spins on “tension free repair as the optimal strategy^19^ and in this regard rigorous research in inguinal surgery has reported that weakness and deficiency lies in anterior abdominal wall, but the price is paid by fascia transversalis to cope with the intra-abdominal pressure which subsequently end up with hernia.^20^ Logically this needs restoration with strengthening of posterior wall^21^. The open tension free polypropylene mesh repair (Lichtenstein) it is contradicted with randomized trial demonstrating no benefits of laparoscopic and open mesh repairs in comparison to mesh-less surgery.^21^ Hence this study was planned to compare the two common techniques (Lichtenstein V/S Darn) and report our experience.

In this study, the peak age for inguinal hernia in both groups were from 20-48 years, which is similar to that reported in studies from Pakistan.^22^ We found no haematoma or seroma in both groups, but the wound infections, prevalence as superficial and deep infections in Group-A and B was 8.6% and 4% respectively, which were resolved through conservative management. Shillcutt et al found 4.4% haematoma and 1.7% wound infections in their study,^23^ while medical literature is replete with different studies each having different rates of haematoma and infections.^6^,^24^ The length of hospital stay was two to six days and one to three days in Group- A & Group-B respectively which is also similar to other studies.^19^,^23^

The time taken to return to daily activities was higher in Group-A as compared to Grou-B. This may be because those patient who had mesh repair, experience more pain and for longer duration.^25^ We have reported recurrence in two different procedures and found to have (2%) in Lichtenstein and (6.5%) in simple Darn repair. The higher rates of recurrence in repair may be because it takes more operative time, there are more chances for injury to vessels and ilio-inguinal nerve in comparison to Lichtenstein repair. These results also correspond to other studies.^21^,^23^ The design of this clinical trial was rational and well executed to extract the logical end points between two procedures, hence our findings are comparable with other series.

## CONCLUSION

We have found that Lichtenstein repair for inguinal hernia is more promising in comparison to Darn repair. However, the hospital stay was higher in this group. Superficial surgical infections were also a bit higher in this group of patients.

### Authors’ Contribution

**GAM:** Conceived, designed and did statistical analysis & editing of manuscript.

**SKAS:** Did data collection and manuscript writing.

**HUR:** Ensured all aspects of study in terms of accuracy and integrity in every aspect of this work with investigations to have a logical substantial inference.

## References

[1] Nicks BA, Askew K (2010). Hernias. eMedicine [online database].

[2] Inguinal hernia: epidemiology [online database] (2010). https://online.epocrates.com/noFrame/showPage.do?method=diseases&MonographId=723&ActiveSectionId=23.

[3] Society of American Gastrointestinal Endoscopic Surgeons www.sages.org.

[4] Zhao G, Gao P, Ma B, Tian J, Yang K (2009). Open mesh techniques for inguinal hernia repair: a meta-analysis of
randomized controlled trials. Ann Surg.

[5] Junge K, Binnebösel M, Rosch R, Öttinger A, Stumpf M, Mühlenbruch G (2008). Influence of mesh materials on the integrity of the vas deferens
following Lichtenstein hernioplasty: an experimental model. Hernia.

[6] Lichtenstein IL, Shulman AG, Amid PK, Montllor MM (1989). The tension-free hernioplasty. Am J Surg.

[7] Matyja A, Kibil W, Pach R, Solecki R, Kulig J, Kamtoh G (2010). Assessment of inguinal hernia treatment results in patients operated
on with mesh using Lichtenstein, PHS and Robbins-Rutkow techniques. Video surgery Miniinv.

[8] Battocchio F, Terranova O, De Santis L (2004). Grande Atlante di Tecnica Chirurgica: Chirurgia Delle
Ernie. UTET Scienze Mediche, Torino.

[9] Adesunkanmi AR, Badmus TA, Ogundoyin O (2004). Determinants of outcome of inguinal herniorrhaphy in Nigerian
patients. Ann Coll Surg Hong Kong.

[10] Sajid MS, Leaver C, Baig MK, Sains P (2012). Systematic review and meta-analysis of the use of lightweight versus
heavyweight mesh in open inguinal hernia repair. Br J Surg.

[11] Tyrell J, Silberman H, Chandrasoma P, Niland J, Shull J (1989). Absorbable versus permanent mesh in abdominal
operations. Surg Gynecol Obstet.

[12] Ansaloni L, Catena F, Coccolini F, Gazzotti F, D’Alessandro L, Pinna AD (2009). Absorbable versus permanent mesh in abdominal
operations. Am J Surg.

[13] Amid PK, Shulman AG (1996). Open 𠇌tension-free” repair of inguinal hernias: the
Lichtenstein technique. Euro J Surg.

[14] Kark AE, Kurzer MN, Belsham PA (1998). Three thousand one hundred seventy-five primary inguinal hernia
repairs: advantages of ambulatory open mesh repair using local
anesthesia. J Am Coll Surg.

[15] Post S, Weiss B, Willer M, Neufang T, Lorenz D (2004). Randomized clinical trial of lightweight composite mesh for
Lichtenstein inguinal hernia repair. Br J Surg.

[16] Breuing K, Butler CE, Ferzoco S, Franz M, Hultman CS, Kilbridge JF, Ventral Hernia Working Group (2010). Incisional ventral hernias: review of the literature and
recommendations regarding the grading and technique of repair. Surgery.

[17] Eker HH, Langeveld HR, Klitsie PJ, van’t Riet M, Stassen LP, Weidema WF (2012). Randomized clinical trial of total extra peritoneal inguinal
hernioplasty vs Lichtenstein repair: a long-term follow-up study. Arch Surg.

[18] Khan M, Mufti TS (1982). A study of incidence of external hernias in NWFP. J Pak Med Assoc.

[19] Pokorny H, Klingler A, Schmid T, Fortelny R, Hollinsky C, Kawji R (2008). Recurrence and complications after laparoscopic versus open inguinal
hernia repair: results of a prospective randomized multicenter
trial. Hernia.

[20] Bendavid R (1989). New techniques in hernia repair. World J Surg.

[21] Bhopal FG, Niazi GH, Iqbal M (1998). Evaluation of Lichtenstein repair for morbidity and
recurrence. J Surg Pak.

[22] Macfarlane Da Thomas LP (1988). Text Book of Surgery.

[23] Rasool MI, Idress A, Qayyum F (1992). Inguinal hernia clinical presentation. Rawal Med J.

[24] Shillcutt SD, Clarke MG, Kingsnorth AN (2010). Cost-effectiveness of groin hernia surgery in the Western Region of
Ghana. Arch Surg.

[25] Stephenson BM, Kingsnorth AN (2011). Inguinal hernioplasty using mosquito net mesh in low income countries
an alternative and cost effective prosthesis. Christmas;Surgery. BMJ.

